# Persistent Neuroadaptations in the Nucleus Accumbens Core Accompany Incubation of Methamphetamine Craving in Male and Female Rats

**DOI:** 10.1523/ENEURO.0480-22.2023

**Published:** 2023-03-09

**Authors:** Jonathan R. Funke, Eun-Kyung Hwang, Amanda M. Wunsch, Raines Baker, Kimberley A. Engeln, Conor H. Murray, Mike Milovanovic, Aaron J. Caccamise, Marina E. Wolf

**Affiliations:** 1 Department of Behavioral Neuroscience, Oregon Health & Science University, Portland, OR 97212; 2 Department of Neuroscience, Rosalind Franklin University of Medicine and Science, North Chicago, IL 60064

**Keywords:** incubation of craving, methamphetamine, nucleus accumbens, rat, sex differences, synaptic plasticity

## Abstract

Relapse is a major problem in treating methamphetamine use disorder. “Incubation of craving” during abstinence is a rat model for persistence of vulnerability to craving and relapse. While methamphetamine incubation has previously been demonstrated in male and female rats, it has not been demonstrated after withdrawal periods greater than 51 d and most mechanistic work used males. Here, we address both gaps. First, although methamphetamine intake was higher in males during self-administration training (6 h/d × 10 d), incubation was similar in males and females, with “incubated” craving persisting through withdrawal day (WD)100. Second, using whole-cell patch-clamp recordings in medium spiny neurons (MSNs) of the nucleus accumbens (NAc) core, we assessed synaptic levels of calcium-permeable AMPA receptors (CP-AMPARs), as their elevation is required for expression of incubation in males. In both sexes, compared with saline-self-administering controls, CP-AMPAR levels were significantly higher in methamphetamine rats across withdrawal, although this was less pronounced in WD100–135 rats than WD15–35 or WD40–75 methamphetamine rats. We also examined membrane properties and NMDA receptor (NMDAR) transmission. In saline controls, MSNs from males exhibited lower excitability than females. This difference was eliminated after incubation because of increased excitability of MSNs from males. NMDAR transmission did not differ between sexes and was not altered after incubation. In conclusion, incubation persists for longer than previously described and equally persistent CP-AMPAR plasticity in NAc core occurs in both sexes. Thus, abstinence-related synaptic plasticity in NAc is similar in males and females although other methamphetamine-related behaviors and neuroadaptations show differences.

## Significance Statement

To study the persistence of vulnerability to methamphetamine craving and relapse during abstinence, we use the “incubation of craving” model. While incubation of methamphetamine craving has been demonstrated previously in male and female rats, most mechanistic work has used male rats and incubation has only been demonstrated through 51 d of abstinence. Here, we show that incubation of methamphetamine craving persists in both sexes for at least 100 d and that an underlying mechanism previously described in males [elevation of synaptic calcium-permeable AMPA receptors (CP-AMPARs) in medium spiny neurons (MSNs) of the nucleus accumbens (NAc) core] occurs in females as well and persists alongside incubation. Thus, a relatively limited period of methamphetamine experience produces extremely long-lasting vulnerability to craving and relapse.

## Introduction

Methamphetamine is a widely abused drug, with relapse occurring in 75% of users within five years after treatment (Brecht and Herbeck, 2014). We investigate the synaptic plasticity that maintains vulnerability to relapse during abstinence using the incubation of craving model, in which cue-induced craving progressively intensifies during abstinence from drug self-administration and then remains high for weeks to months (Pickens et al., 2011; Altshuler et al., 2020; Fredriksson et al., 2021). Incubation of craving also occurs in humans after discontinuing use of methamphetamine (G. Wang et al., 2013) and other drugs (Li et al., 2016).

As aspects of addiction differ for men and women, it is important to compare the sexes in animal models (Sanchis-Segura and Becker, 2016). Female rats more readily acquire methamphetamine self-administration and exhibit faster escalation of intake, higher motivation in progressive ratio tests, and greater reinstatement of methamphetamine seeking following extinction training (Roth and Carroll, 2004; Holtz et al., 2012; Reichel et al., 2012; Cox et al., 2013). In contrast, incubation of methamphetamine craving does not show sex differences (Venniro et al., 2017, 2018; Daiwile et al., 2019, 2021; Everett et al., 2020). Thus, abstinence-related plasticity may be similar between male and female rats.

Similar to preclinical models, human studies indicate sex differences in methamphetamine use. For example, different factors motivate men and women to take methamphetamine and they experience different problems resulting from methamphetamine dependence (Brecht et al., 2004). However, in studies of craving or relapse, clear sex differences do not emerge, suggesting convergence with rat data in the incubation model. Thus, one study of abstinent methamphetamine users found slightly lower mean craving scores in women (Galloway et al., 2010), while another found no sex differences in the ability of methamphetamine-related cues to elicit craving or physiological responses in methamphetamine-dependent subjects (Tolliver et al., 2010). Regarding relapse, one study found that women were less able to remain abstinent during treatment but sex did not predict the pattern of methamphetamine use at discharge, six or 12 months after admission (Hillhouse et al., 2007). Likewise, sex did not predict relapse within six months of treatment completion (Brecht et al., 2004) or relapse status (He et al., 2013). However, another study found that male sex was associated with a greater likelihood of reporting past-month methamphetamine use at one and five years postbaseline (Lanyon et al., 2019).

So far, comparisons of methamphetamine incubation in male and female rats (above) have been restricted to the first month of withdrawal and even in males the longest withdrawal examined was 51 d (Shepard et al., 2004). Some incubation-related neurobiological endpoints have been compared between the sexes, including gene expression and dopamine metabolism (Daiwile et al., 2019, 2021, 2022; Miller et al., 2022). However, an adaptation required for expression of methamphetamine incubation in male rats after forced abstinence, namely strengthening of AMPA receptor (AMPAR) transmission in medium spiny neurons (MSN) in nucleus accumbens (NAc) core through synaptic incorporation of Ca^2+^-permeable AMPARs (CP-AMPAR; Scheyer et al., 2016), has not been studied in females.

To address these gaps, we assessed cue-induced seeking and electrophysiological properties of NAc core MSNs over ∼100 d of forced abstinence from methamphetamine self-administration in male and female rats. In addition to extending our studies of CP-AMPAR transmission to females and later withdrawal times, the present study is the first to examine membrane properties and NMDA receptor (NMDAR) transmission in the NAc after incubation of methamphetamine craving in either sex. We focused on the NAc core because it is required for incubated methamphetamine seeking after voluntary abstinence in males [(Rossi et al., 2020); the shell subregion was not required] and for cued-methamphetamine seeking in extinction-reinstatement models and a different abstinence model (Hiranita et al., 2006; Rocha and Kalivas, 2010; Kearns et al., 2022).

## Materials and Methods

### Subjects and surgery

All procedures were approved by the Oregon Health & Science University or the Rosalind Franklin University of Medicine and Science Institutional Animal Care and Use Committees in accordance with the United States Public Health Service *Guide for Care and Use of Laboratory Animals*. Adult male (250–275 g) and female (225–250 g) Sprague Dawley rats (Envigo) were housed three to four per cage under a reverse 12/12 h light/dark cycle with food and water provided *ad libitum*. Rats acclimated to the colony for a minimum of 5 d preceding intravenous catheter surgery. Rats were anesthetized with ketamine-xylazine (80–10 mg/kg, i.p., respectively; MWI Animal Health) or isoflurane (MWI Animal Health). A SILASTIC tubing (The Dow Chemical Company) catheter (Plastics One) was inserted into the right or left jugular vein. Banamine (2.5 mg/kg, i.m.; MWI Animal Health) or meloxicam (5 mg/kg, s.c.; Covetrus) was administered as an analgesic preoperatively and postoperatively. Following catheter surgery, rats were single-housed with enrichment (nylabones and nesting packs) for the duration of the experiment. During recovery from surgery (5–7 d) and the first 9 d of methamphetamine self-administration, catheters were flushed every 24–48 h with 0.9% sterile saline (Baxter International) containing the β-lactam antibiotic cefazolin (30 mg, i.v.; Covetrus). Prior studies have shown that the β-lactam antibiotic ceftriaxone (typically administered at 200 mg/kg, i.p., for 5–12 d), increases expression of the glutamate transporter GLT-1 and normalizes behavioral and neurochemical effects produced by repeated administration of drugs of abuse (Smaga et al., 2020). In addition to using a different antibiotic, our studies employed a lower dose, a different route of administration (intravenous vs intraperitoneal), and a greater delay between the last antibiotic dose and the experimental procedure (2–100+ d in our studies vs 1 d in many prior ceftriaxone studies). These features of our design, combined the fact that saline controls received the same cefazolin regimen as methamphetamine rats, reduce concerns about an effect of cefazolin on our experimental results although some influence cannot be completely ruled out.

### Drug self-administration

Rats were trained to self-administer methamphetamine or saline (control condition) as described previously (Scheyer et al., 2016; Murray et al., 2019, 2021). Training consisted of 10 sessions, each lasting 6 h, conducted over 12 d with 2 d off (after the fourth and seventh days of training). Sessions began 1–4 h after the beginning of the dark cycle. Rats self-administered methamphetamine (in 0.9% saline) at a dose of 0.1 mg/kg/infusion (0.065 ml/infusion) under a fixed-ratio-1 reinforcement schedule. Control rats self-administered saline (0.065 ml/infusion) under an identical schedule. Self-administration was performed in operant chambers equipped with two nose-poke holes. Active hole responses activated the infusion pump and led to the presentation of a 20-s light cue (yellow light illuminating the active hole). Each infusion was followed by a 20-s timeout period that corresponded to the 20-s light cue presentation during which nose pokes were recorded but did not induce drug delivery. Nose pokes in the inactive hole had no consequences. During withdrawal/forced abstinence (these terms will be used interchangeably), rats remained single housed in their home cages.

### Tests for cue-induced methamphetamine seeking

Each methamphetamine rat received a single cue-induced seeking test on withdrawal day (WD)1, WD7, WD30, or WD100. For the seeking test, rats were returned to their self-administration chambers and tested for 30 min under extinction conditions. Thus, during the seeking test, a nose poke in the active hole resulted in presentation of a 20-s light cue (the same cue previously paired with methamphetamine infusion) but no methamphetamine infusion was given. There was no time-out period implemented during the seeking test (i.e., nose pokes during the 20-s light cue were counted). The number of responses in the active hole was used as a measure of methamphetamine seeking or craving. Responses in the inactive hole were recorded but had no consequences.

### Estrous cycle classification

A published protocol was followed (Becker et al., 2005). Briefly, every female rat was vaginally swabbed with a cotton tipped applicator (Puritan Medical Products Company) dampened with warm sterile 0.9% saline. Swabs were smeared onto Superfrost Plus White Micro Slides (VWR International), and stained using Toludine Blue (Sigma-Aldrich). Female rats were swabbed 2–3 h after their seeking test, and daily for 2–3 d afterward. Male rats were subjected to a similar procedure (swabbing of genital area) following an identical schedule. Estrous stage was classified as either vaginal estrous or nonestrous, with vaginal estrus identified by a proliferation of cornified epithelial cells.

### Whole-cell patch-clamp recordings

A subset of rats was used for electrophysiological recordings at least one week after their single drug seeking test (for more information, see Experimental design and statistical analyses). Saline rats were all recorded after WD30 but were not grouped by withdrawal period because we have never observed time-dependent changes in MSN properties in saline rats recorded between WD1 and ∼WD70 over >10 years of recordings. All electrophysiological procedures were adapted from those previously described (Conrad et al., 2008; McCutcheon et al., 2011; Scheyer et al., 2016; Murray et al., 2021). Briefly, rats were anesthetized with chloral hydrate (400–600 mg/kg, i.p.) and brains were rapidly removed. Coronal slices at the level of the NAc (280 μm) were cut with a vibrating microtome in ice-cold *N*-methyl-D-glucamine (NMDG)-based cutting solution and then transferred to warm (32–34°C) artificial CSF (aCSF) containing (in mm): 93 NMDG, 2.5 KCl, 1.2 NaH_2_PO_4_, 30 NaHCO_3_, 20 HEPES, 25 glucose, 5 sodium ascorbate, 3 sodium pyruvate, 10 MgCl_2_, 0.5 CaCl_2_ (oxygenated with 95% O_2_/5% CO_2_, pH 7.3, 300–310 mOsm). Slices were incubated in this solution at 32–34°C for 15 min and then transferred to a holding chamber containing modified aCSF (in mm): 109 NaCl, 2.5 KCl, 1 MgCl_2_, 2.5 CaCl_2_ 1.2 NaH_2_PO_4_, 35 NaHCO_3_, 11 glucose, 20 HEPES, 0.4 sodium ascorbate (pH 7.3, 300–310 mOsm) at room temperature. Whole-cell patch-clamp recordings were conducted at least 1 h after slicing in oxygenated (95% O_2_/5% CO_2_) and warm (30–32°C) aCSF (in mm): 126 NaCl, 3 KCl, 1.5 MgCl_2_, 2.4 CaCl_2_, 1.2 NaH_2_PO_4_, 11 glucose, 26 NaHCO_3_. Picrotoxin (0.1 mm) and APV [(2R)-amino-5-phosphonopentanoate; 0.05 mm] were added into the recording aCSF to pharmacologically isolate AMPAR transmission. NMDAR-mediated currents were measured in the presence of picrotoxin and CNQX (0.02 mm). Voltage clamp recordings were conducted using patch pipettes (2–3 MΩ) filled with a Cs-based/spermine-containing internal solution (in mm): 115 CsMeSO_3_, 10 HEPES, 20 CsCl, 0.6 EGTA, 7.5 QX-314, 10 NaPhosphocreatine, 4 MgATP, 0.4 NaGTP, 0.1 spermine; adjusted to pH 7.3 and 290 mOsm. The liquid junction potential (∼13 mV) for CsMeSO_3_ recordings was corrected for current–voltage (I–V) relationship measurements. The reversal potentials were stable (±2 mV) on different days and corrected to 0 mV. Spermine (100 μm) was added fresh to the internal solution each day. A bipolar tungsten stimulating electrode (FHC) placed ∼200 μm from the recording site was used to elicit EPSCs in MSNs. Only neurons that exhibited stable baseline synaptic responses (<15% variability, 15 min) were included. For current clamp recordings, recording pipettes were filled with K-gluconate based internal solution (in mm): 140 K-gluconate, 10 HEPES, 0.2 EGTA, 2 MgCl_2_, 0.1 CaCl_2_, 4 Na_2_-ATP, 0.3 Na-GTP; adjusted to pH 7.3 and 290 mOsm. The intrinsic excitability evaluations were performed at −80 mV by adjusting membrane potential (typically less than ±50-pA current injection) in current clamp mode. For analysis of excitability measurements, each protocol was repeated three times and averaged.

### Analysis of surface-expressed and total NMDAR subunits

**Figure 1.**
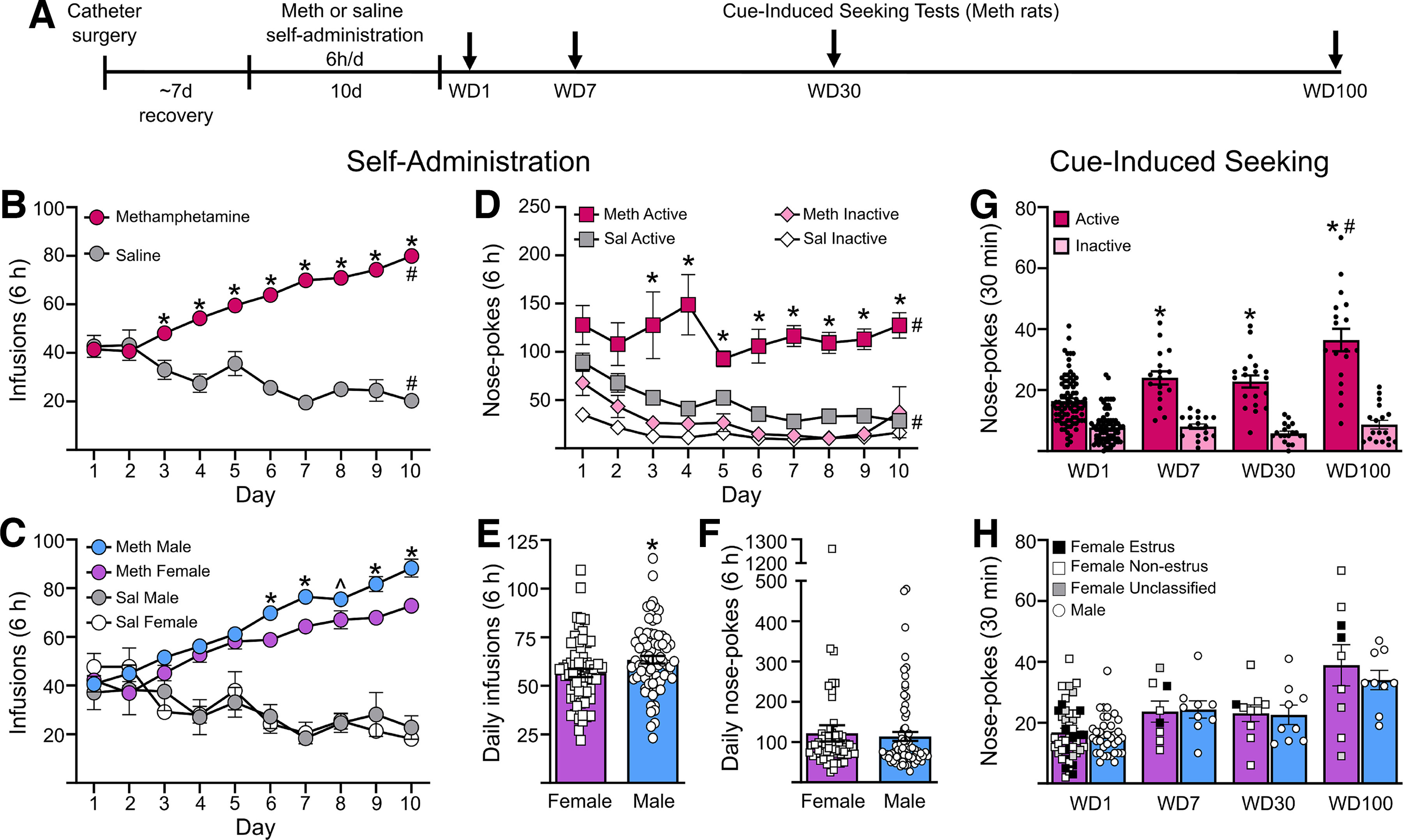
Methamphetamine self-administration and incubation of craving in male and female rats. ***A***, Rats were trained to self-administer methamphetamine (Meth) or saline (Sal) for 6 h/d for 10 d. Each Meth rat underwent a single cue-induced seeking test on either withdrawal day (WD) 1, WD7, WD30, or WD100. At least a week after the test, many of the Meth rats, along with the saline rats, were used for electrophysiological recordings. ***B***, Average infusions during self-administration training for methamphetamine (*n* = 136) and saline (*n* = 17) rats. *p < 0.05 compared with saline rats; #*p* < 0.05 day 10 compared with day 1. ***C***, Number of infusions across all self-administration sessions for male and female rats. Male rats took a significantly higher number of infusions than female rats on days 6, 7, 9, and 10 of self-administration, and trended toward higher intake on day 8. *p < 0.05, ^*p* = 0.051. ***D***, Active and inactive nose pokes during self-administration for methamphetamine and saline rats. Methamphetamine rats performed a significantly higher number of nose-pokes in the active port than saline rats. *p < 0.05. Nose-pokes in the active port were significantly higher compared with inactive port nose pokes in both methamphetamine and saline rats on all days of self-administration. #*p* < 0.05. ***E***, Average daily intake of methamphetamine was higher in male rats (*n* = 63) compared with female rats (*n* = 73). *p < 0.05. ***F***, Average daily nose-pokes in the active port was similar in male and female rats self-administering methamphetamine. ***G***, During seeking tests, methamphetamine rats had a significantly higher number of nose pokes in the previously active hole on WD7, WD30, and WD100 compared with WD1. Rats on WD100 had significantly higher nose pokes in the previously active hole compared with WD7 and WD30. *p < 0.05 compared with WD1, #*p* < 0.05 compared with WD7 and WD30. Group sizes: WD1 (*n* = 46 females, *n* = 36 males), WD7 (*n* = 8 females, *n* = 9 males), WD30 (*n* = 10 females, *n* = 9 males), WD100 (*n* = 9 females, *n* = 9 males). ***H***, There were no differences in the number of nose pokes in the previously active hole between male and female rats. Estrous cycle stage was determined by vaginal swabbing following cue-induced seeking tests with the goal of comparing estrous and nonestrous females. However, there were not enough females in estrus to make a reliable assessment. In lieu of statistical analysis, females in estrus on the day of their seeking test are identified by filled black squares, females not in estrus are indicated by white squares, un-classified females are denoted by gray squares, and males are indicated by white circles. Data are presented as mean ± SEM, with individual data points on all bar graphs.

We used biotinylated NAc core tissue from a cohort of male rats, prepared approximately two years before the main cohort shown in Figure 1, that self-administered saline or methamphetamine under identical conditions and were killed on WD21 or WD48 without receiving a drug seeking test. Only males were run because the grant supporting the work at the time did not include funds to study both sexes. Some aliquots of tissue from these saline and methamphetamine rats were used to measure different proteins in experiments previously published as part of separate studies (Murray et al., 2019, 2021). Their self-administration training data, which are similar to those shown in Figure 1, were included in one of these publications (Murray et al., 2021). Here, different aliquots were used to measure NMDAR subunits. Briefly, bilateral NAc core tissue was dissected using a 1.5-mm biopsy punch from two 1-mm-thick slices prepared with a brain matrix. NAc samples were biotinylated and, after reserving an aliquot of homogenate for measurement of total protein levels, samples were processed to separate biotinylated proteins bound to NeutrAvidin beads (bound material, surface-expressed proteins) from the nonbiotinylated (unbound) material, as detailed previously (Ferrario et al., 2011b; Loweth et al., 2014; Murray et al., 2021). Aliquots of homogenates (total protein) and biotinylated fractions (surface protein) were stored at −80°C.

### Analysis of NMDAR subunits in postsynaptic density (PSD) fractions

We used NAc core PSD fractions from a cohort of male rats, prepared approximately two years before the main cohort shown in Figure 1, that self-administered saline or methamphetamine under identical conditions and were killed on WD61 without receiving a drug seeking test. Only males were run because the grant supporting the work at the time did not include funds to study both sexes. No previous results obtained using tissue from this cohort of rats have been published. Their self-administration training data are very similar to data from the male rats presented in Figure 1 and our published data (Scheyer et al., 2016; Murray et al., 2019, 2021). For example, the number of infusions taken by these rats did not differ from male methamphetamine rats presented in Figure 1*C*,*E* (generalized linear model; no significant between subject experiment × within subject day interaction, *p* > 0.05; no main effect of experiment, *p* > 0.05; significant main effect of day, *F*_(2.503,172.4)_ = 20.98, *p* < 0.0001). Briefly, bilateral NAc tissue was punched from a 2-mm-thick slice prepared with a brain matrix. Punches were centered on the NAc core region but also contained tissue from NAc shell. To obtain a Triton-insoluble PSD fraction, we used a previously described subcellular fractionation procedure (Davies et al., 2007, 2008; Goebel-Goody et al., 2009) that we have validated in our laboratory (Ferrario et al., 2011a). Each NAc sample was homogenized in 3 ml of sucrose homogenization buffer (10 mm HEPES, 0.32 m sucrose, 5 mm NaF, 1 mm NaVO, 2 mm EDTA, pH 7.4) in a glass grinding vessel with a rotating Teflon pestle (Wheaton Overhead Stirrer; 3000 RPM for 12 passes). The homogenate was centrifuged (800 × *g*, 10 min, 4°C) to remove the pelleted nuclear fraction (P1). The resulting supernatant (S1) was centrifuged (10,000 × *g*, 15 min, 4°C) to yield a crude membrane fraction (P2). The P2 fraction was washed twice with sucrose homogenization buffer and re-suspended in 4 ml of HEPES-buffered sucrose containing Triton X-100 (0.5% v/v) using a motorized pellet pestle mixing/grinding rod (Kontes). The suspension was then incubated with gentle rotation (20 min, 4°C) and centrifuged (32,000 × *g*, 20 min) to yield the insoluble pellet (PSD fraction), which was washed twice before use. The PSD fraction was re-suspended in Laemmli sample treatment buffer containing 100 mm dithiothreitol (DTT) and stored at −20°C.

### Immunoblotting

Samples were processed for immunoblotting as described previously (Conrad et al., 2008; Ferrario et al., 2011b; Loweth et al., 2014; Murray et al., 2019, 2021). Loading was determined by a protein assay. Samples were run on 4–12% Bis-Tris gels (Bio-Rad) and transferred to PVDF membranes. Membranes were blocked at room temperature [5% evaporated milk, 1% normal goat serum in tris-buffered saline (TBS) containing 0.05% Tween (TBS-T)], incubated at 4°C overnight in primary antibodies diluted in TBS-T, rinsed in TBS-T, incubated in secondary antibodies diluted in TBS-T for 2 h at room temperature, and then immersed in chemiluminescence (ECL) detecting substrate (GE Healthcare). Images of immunoblots were taken using an Amersham Imager 600 (GE Healthcare) and quantified using ImageQuant TL software (GE Healthcare). A rolling ball analysis was used and densities for bands of interest in each lane were determined. For total homogenate samples, GAPDH was used as a loading control; for immunoblotting of surface-expressed (biotinylated) fractions, we have not identified a surface-expressed protein that we are confident using as a control and so we load carefully based on protein assay data. The same applies to PSD fractions. Data are presented as mean ± SEM expressed as percent of control (rats that self-administered saline). For some proteins, we were unable to quantify immunoreactivity for some lanes because of an unclear band, smudge on the blot, or air bubble obstructing the signal. However, from experimental groups of 8–12 rats, we lost at most one to two samples/group and after these losses we still had 8–10 rats/group. No replication of blots was performed. The following primary antibodies were used for analysis of biotinylation experiments (starting material and biotinylated fraction) and PSD fractions, with studies validating antibody specificity listed in parentheses after each antibody: GluN1 (1:500) rabbit, Cell Signaling Technology, 5704 (Y. Wang et al., 2015; Cell Signaling Technology Product Information); GluN2A (1:1000), rabbit, Novus Biologicals, NB300-105 (Novus Product Datasheet, v20.1 Updated 9/14/16); GluN2B (1:1000), rabbit, Cell Signaling Technology, 14 544 (Pietrogrande et al., 2019; Yang et al., 2020); GluN3A (1:200), rabbit, OriGene, TA328843 (OriGene Product Information); GluN3B (1:200), rabbit, OriGene, TA328844 (OriGene Product Information), and GAPDH (1:5000), mouse, Calbiochem, CB1001. The following horseradish peroxidase conjugated secondary antibodies were used: goat anti-rabbit IgG (H+L; 1:10;000), Invitrogen, G21234 or goat anti-mouse IgG (H+L; 1:10,000), Invitrogen, G21040.

### Experimental design and statistical analyses

All rats self-administered saline or methamphetamine and underwent different periods of withdrawal. Rats destined for biochemical analysis did not receive seeking tests. Other rats received a single cue-induced seeking test on WD1, WD7, WD30, or WD100. A subset of these rats was randomly selected for electrophysiological analysis based on the recording schedule. All data are visualized as mean ± SEM. Significance was set at *p* < 0.05. Parametric assumptions were analyzed as follows: *F* test for heteroscedasticity, Shapiro–Wilk test of standardized residuals by group for normality, Levene’s test for equality of variance (means-based), and analysis of kurtosis and skewness values. For all experimental groups, n values are provided in figure legends.

#### Behavior

We analyzed self-administration training data (nose-pokes and infusions) and results of cue-induced methamphetamine seeking tests (nose-pokes) in male and female rats (Fig. 1). The smallest group size was eight rats. Both self-administration training and cue-induced seeking data were nonparametric. Training infusions were analyzed via generalized linear mixed model, with training day (session) analyzed as a repeated measures (RM) factor, while drug group (methamphetamine vs saline) and sex (male vs female) were between-subject factors. Training nose-pokes were similarly analyzed in a generalized linear mixed model format, with session and nose-pokes (active hole vs inactive hole) as within-subject factors, and sex (male vs female) and drug group (methamphetamine vs saline) as between-subject factors. Cue-induced seeking data were analyzed via a Generalized Linear Model, with withdrawal day and sex (male vs female) as the between-subject factors (F values presented under ANOVA or generalized linear mixed model analyses; Wald χ^2^ values reported for generalized linear model analyses). All *post hoc* analyses were conducted using a sequential Bonferroni correction. Average daily infusions were analyzed with an unpaired *t* test and average daily active nose pokes were analyzed with Mann–Whitney *U* test. See Results and legend to Figure 1 for details of statistical analysis.

#### Electrophysiology

**Figure 2.**
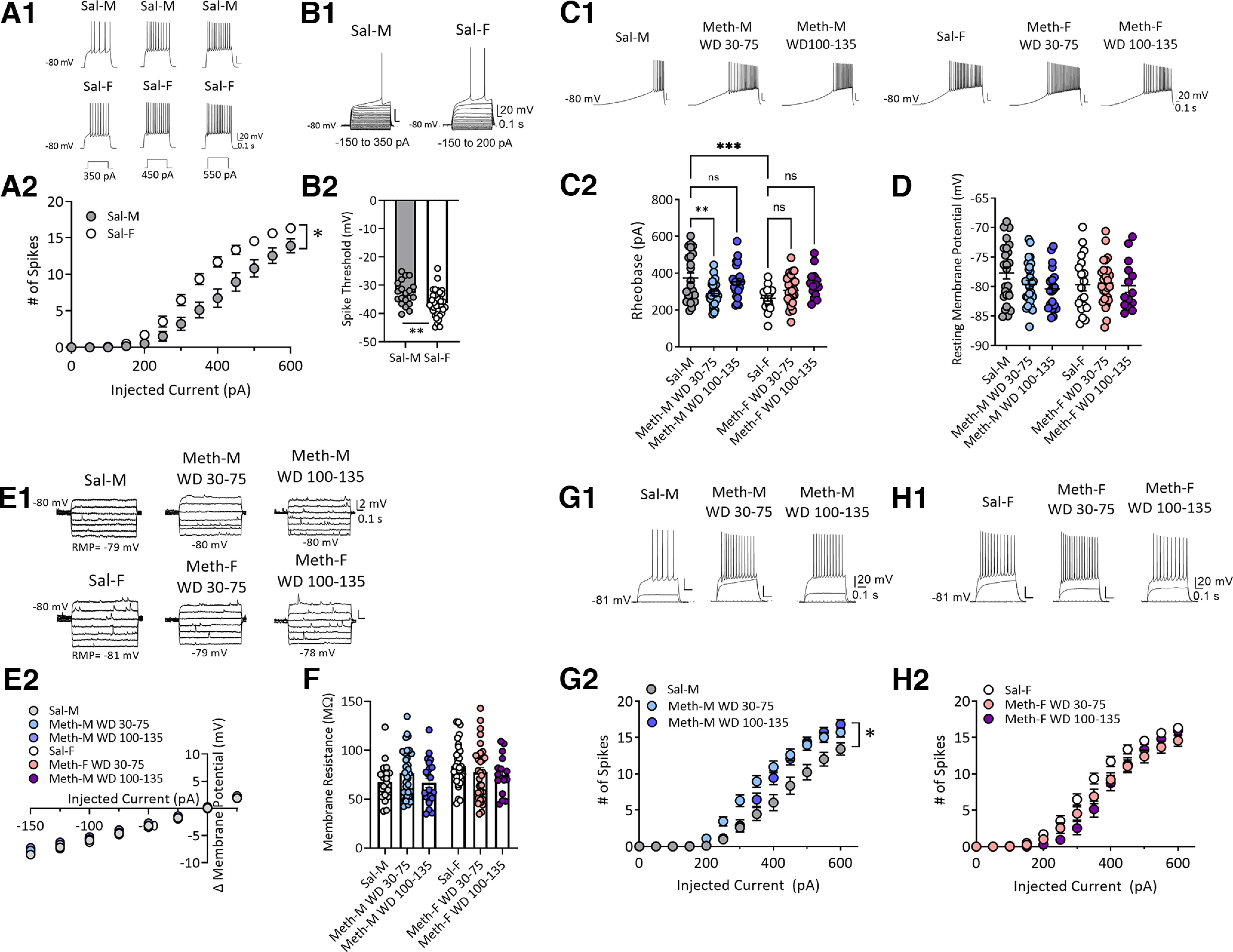
Sex-specific alteration of membrane excitability of NAc core MSNs after withdrawal from long-access methamphetamine self-administration. ***A1***, Representative traces of action potentials in response to depolarizing step current injections (350, 450, and 550 pA) in MSNs from saline male (Sal-M; upper panel) or saline female (Sal-F; lower panel) rats. ***A2***, Mean current–voltage (I–V) relationship of MSNs. Average number of action potentials triggered by incremental depolarizing step currents (0–600 pA, Δ50 pA, 500-ms duration) in Sal-M rats (23 cells, 6 rats) and Sal-F rats (34 cells, 8 rats). MSNs from females showed a significantly higher excitability at current injections from 300 to 550 pA than males. * indicates significant main effects of injected current (*p* < 0.0001) and sex (*p* < 0.01), and a significant injected current × sex interaction (*p* < 0.0001); *post hoc* Bonferroni’s multiple comparisons at 300 pA, *p* < 0.01; 350 pA, *p* < 0.001; 400–450 pA, *p* < 0.0001; 500 pA, *p* < 0.01; and 550 pA, *p* < 0.05. ***B1***, Representative traces. ***B2***, Spike threshold was higher in Sal-M rats (20 cells, 6 rats) compared with Sal-F rats (36 cells, 8 rats; two-tailed unpaired *t* test **p < 0.01). ***C1***, Representative traces of ramp current injection (500 pA/2 s)-induced firing in MSNs. ***C2***, The rheobase current measured by using a ramp current injection in saline rats and each methamphetamine group. A bigger average rheobase current in MSNs from Sal-M rats was observed compared with Sal-F rats. MSNs from the male methamphetamine (Meth-M) WD30–75 group showed lower rheobase currents compared with Sal-M rats with no differences between Sal-M and Meth-M WD100–135. The rheobase currents of MSNs from female methamphetamine rats (Meth-F) were similar in all Meth groups compared with saline controls (Tukey’s test, **p < 0.01, ***p < 0.001; Sal-M, 24 cells, 6 rats; Meth-M WD30–75, 29 cells, 8 rats; Meth-M WD100–135, 22 cells, 6 rats; Sal-F, 19 cells, 6 rats; Meth-F WD30–75, 28 cells, 7 rats; Meth-F WD100–135, 14 cells, 5 rats). ***D***, Average resting membrane potential of MSNs was similar in each group (Tukey’s test, *p* > 0.05; Sal-M, 25 cells, 6 rats; Meth-M WD30–75, 29 cells, 8 rats; Meth-M WD100–135, 21 cells, 6 rats; Sal-F, 19 cells, 6 rats; Meth-F WD30–75, 28 cells, 7 rats; Meth-F WD100–135, 14 cells, 5 rats). ***E1***, Representative membrane voltage traces in response to subthreshold current injections. ***E2***, Mean I–V relationships following incremental subthreshold current injections (step current injections, –150 to 25 pA/Δ25 pA, 1 s). ***F***, Average input membrane resistance (Rm) of MSNs recorded from saline, Meth WD30–75 and Meth WD100–135 groups in males and females was similar (Tukey’s test, *p* > 0.05; Sal-M, 21 cells, 6 rats; Meth-M WD30–75, 32 cells, 8 rats; Meth-M WD100–135, 22 cells, 6 rats; Sal-F, 34 cells, 8 rats; Meth-F WD30–75, 33 cells, 8 rats; Meth-F WD100–135, 16 cells, 6 rats). ***G1***, Representative traces of action potentials evoked by 0-, 200-, 400-pA current steps. ***G2***, Mean number of action potentials in response to depolarizing current injections (step, 0–600 pA/Δ50 pA, 0.5 s) in males. Meth-M WD30–75 rats showed increased action potentials at 300–500 pA, whereas Meth-M WD100–135 rats showed increased action potentials at 500 pA compared with Sal-M rats. * indicates significant main effects of injected current (*p* < 0.0001) and group (*p* < 0.01), and a significant injected current × group (*p* < 0.0001); *post hoc* Tukey’s multiple comparisons, Sal-M versus Meth-M WD30–75: at 300 pA, *p* < 0.05; 350 pA, *p* < 0.001; 400 pA, *p* < 0.0001; 450 pA, *p* < 0.001; and 500 pA, *p* < 0.05; Sal-M versus Meth-M WD100–135: at 500 pA, *p* < 0.05; Sal-M, 22 cells, 6 rats; Meth-M WD30–75, 31 cells, 8 rats; Meth-M WD100–135, 16 cells, 7 rats. ***H1***, Representative traces of action potentials evoked by 0-, 200-, 400-pA current steps. ***H2***, Mean number of action potentials in response to depolarizing currents injected (step, 0–600 pA/Δ50 pA, 0.5 s) in females. No difference was observed between saline and methamphetamine groups (*post hoc* Tukey’s multiple comparisons *p* > 0.05; Sal-F, 34 cells, 8 rats; Meth-F WD30–75, 28 cells, 7 rats; Meth-F WD100–135, 15 cells, 6 rats). Data are presented as mean ± SEM.

**Figure 3.**
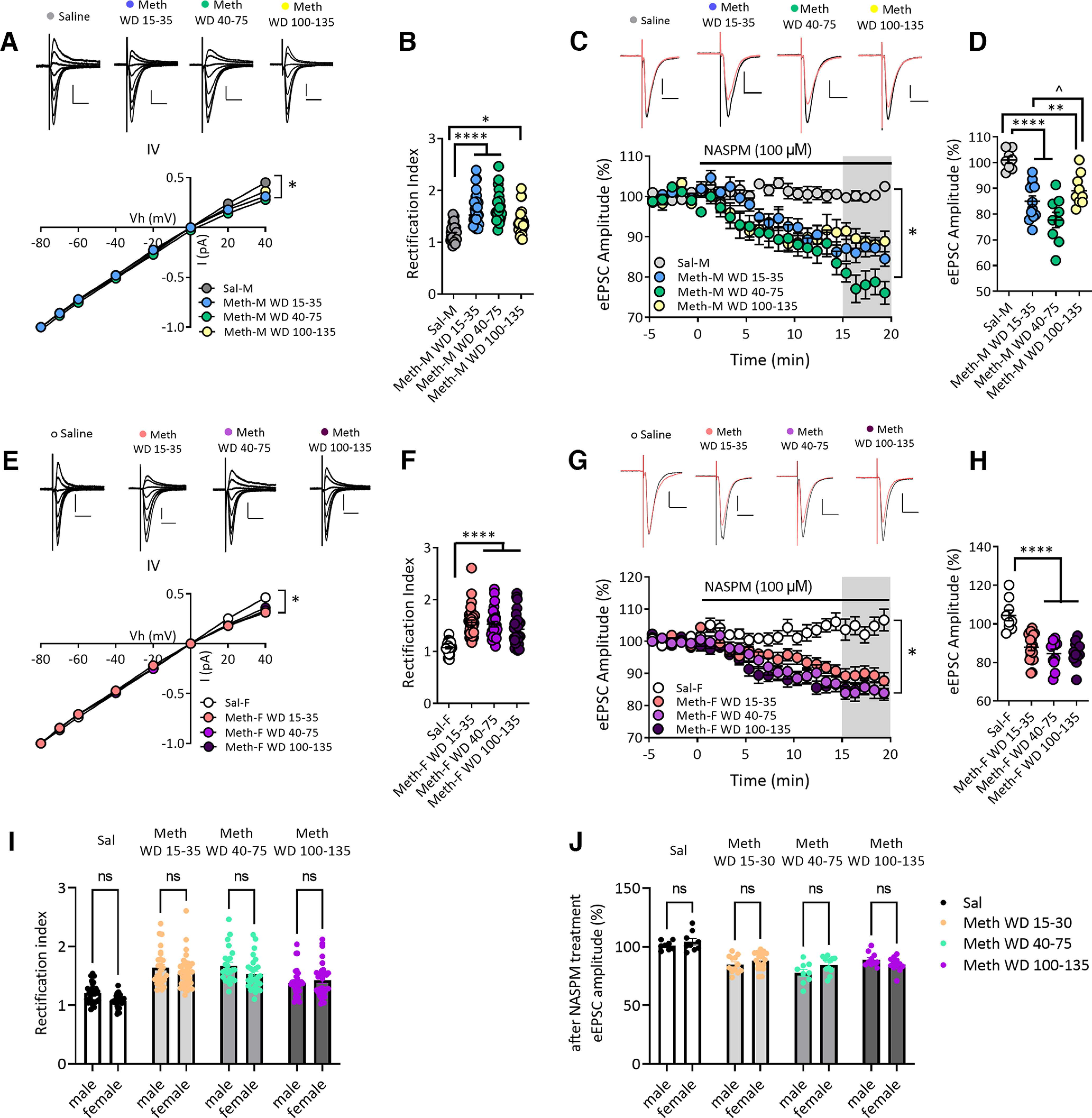
Increased GluA2-lacking AMPARs in NAc core MSNs after withdrawal from long-access methamphetamine self-administration in male and female rats. ***A***, AMPAR-mediated eEPSCs (top) recorded at −80, −70, −60, −40, −20, 0, 20, and 40 mV from NAc core MSNs of male rats after withdrawal from saline or methamphetamine (Meth) self-administration. Scale bar: 200 pA/20 ms. Bottom, I–V plot of AMPAR-mediated synaptic responses of MSNs at different membrane holding potentials. The saline male (Sal-M) group showed a linear I–V relationship, but inward rectifying I–V relationships for methamphetamine male (Meth-M) rats were detected at all withdrawal times. * indicates significant main effects of group and Vh, as well as Vh × group interaction (all *p* < 0.0001). ***B***, Quantification of rectification index or RI [eEPSC_−70mV_/(−70-Erev)]/[eEPSC_+40mV_/(+40-Erev)] showing a higher RI in NAc core MSNs from Meth-M rats compared with Sal-M rats (Tukey’s *post hoc*, Sal-M vs Meth-M WD15–35, ****p < 0.0001; Sal-M vs Meth-M WD40–75, ****p < 0.0001; Sal-M vs Meth-M WD100–135, *p < 0.05; Sal-M, 7 rats, 28 cells; Meth-M WD15–35, 9 rats, 27 cells; Meth-M WD40–75, 8 rats, 24 cells; Meth-M WD100–135, 7 rats, 30 cells). ***C***, Representative eEPSC traces (top: black line, baseline; red line, 15–20 min after NASPM application) and their time courses (bottom) before and during 100 μm NASPM application in Sal-M or Meth-M rats. Scale bar: 200 pA/20 ms. * indicates significant main effects of group (*p* < 0.001) and time (*p* < 0.0001), and a significant time × group interaction (*p* < 0.0001). ***D***, Mean eEPSC amplitudes measured 15–20 min after NASPM application. NASPM decreased eEPSC amplitudes in MSNs from all male Meth groups but not saline rats, indicating upregulation of CP-AMPARs after Meth withdrawal (*post hoc* Tukey’s multiple comparisons, **p < 0.01, ****p < 0.0001 vs Sal-M group; ^*p* < 0.01 Meth-M WD15–35 vs WD100–135; Sal-M, 6 rats, 8 cells; Meth-M WD15–35, 6 rats, 12 cells; Meth-M WD40–75, 6 rats, 9 cells; Meth-M WD100–135, 6 rats, 10 cells). ***E***, Top, AMPAR-mediated eEPSCs recorded from NAc MSNs of saline female (Sal-F) rats or methamphetamine female (Meth-F) rats. Bottom, Rectifying I–V relationships were observed in Meth-F rats at all withdrawal times. * indicates significant main effects of group and Vh and a significant Vh × group interaction (all *p* < 0.0001). Sal-F, *n* = 8 rats, 27 cells; Meth-F WD15–35, *n* = 8 rats, 36 cells; Meth-F WD40–75, *n* = 8 rats, 28 cells; Meth-F WD100–135, *n* = 7 rats, 32 cells. Scale bar: 200 pA/20 ms. ***F***, Increased RI in Meth-F rats compared with Sal-F rats (*post hoc* Tukey’s multiple comparisons, ****p < 0.0001 all Meth groups vs Sal-F; Sal-F, 8 rats, 27 cells; Meth-F WD15–35, 8 rats, 36 cells; Meth-F WD 40–75, 8 rats, 28 cells; Meth-F WD100–135, 7 rats, 32 cells). ***G***, Example eEPSCs (top: black line, baseline; red line, 15–20 min after NASPM application) recorded from NAc core MSNs of female rats and time courses (bottom) before and during NASPM application. Scale bar: 200 pA/20 ms. * indicates significant main effects of group and time, and a significant group × time interaction (all *p* < 0.0001). Sal-F, 5 rats, 9 cells; Meth-F WD15–35, 8 rats, 18 cells; Meth-F WD 40–75, 6 rats, 11 cells; Meth-F WD100–135, 6 rats, 12 cells. ***H***, Mean eEPSC amplitudes measured 15–20 min after NASPM application showing that the sensitivity to NASPM was increased after withdrawal from Meth in female rats (*post hoc* Tukey’s multiple comparisons, ****p < 0.0001 all Meth-F groups vs Sal-F). Data are presented as mean ± SEM. ***I***, Bar graphs showing no differences in RI values between males and females in the saline group and males and females in each of the methamphetamine groups (all *p* > 0.05). ***J***, The effect of NASPM on eEPSC amplitudes after 15- to 20-min bath application was similar between males and females in the saline group and males and females in each of the methamphetamine groups (all *p* > 0.05). Data shown in panels ***I*** and ***J*** are replotted from ***B*** and ***D*** (male rats) and ***F*** and ***H*** (female rats) for ease of comparison.

**Figure 4.**
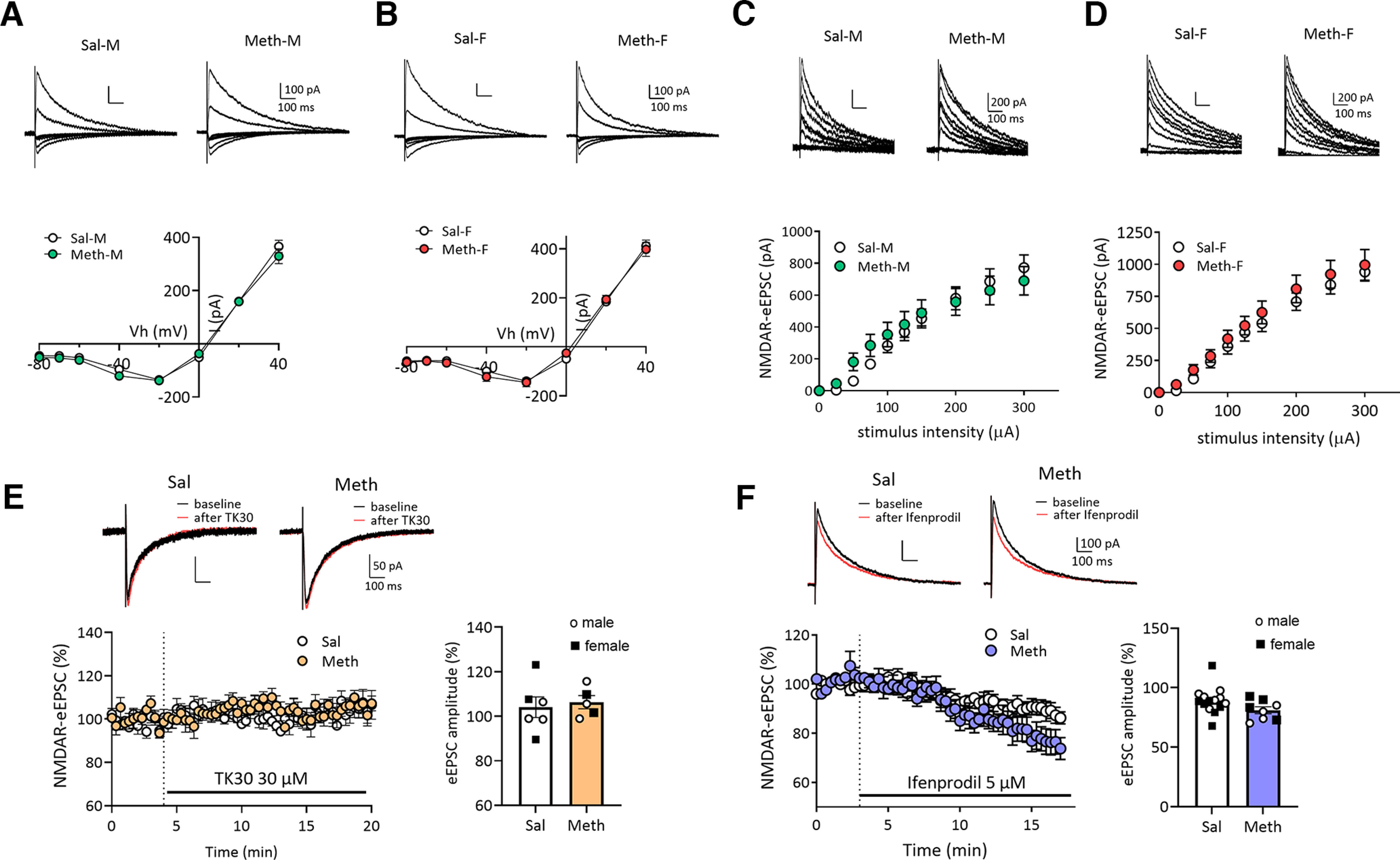
No alteration in NMDAR-mediated synaptic transmission in NAc core MSNs after withdrawal from long-access methamphetamine self-administration in male and female rats. ***A***, ***B***, Representative traces (top) of NMDAR-eEPSCs and I–V plot (bottom) of NMDAR-mediated eEPSCs at different membrane holding potentials (−80, −70, −60, −40, −20, 0, 20, 40 mV) in NAc core MSNs in male (M) or female (F) rats after saline (Sal) or methamphetamine (Meth) withdrawal. There were no significant differences in the voltage-dependent properties of NMDAR-mediated eEPSCs between saline and methamphetamine rats in males (***A***, Bonferroni test, *p* > 0.05; Meth-M, *n* = 8 rats, 19 cells; Sal-M, *n* = 5 rats, 20 cells) or in females (***B***, Bonferroni test, *p* > 0.05; Meth-F, *n* = 7 rats, 11 cells; Sal-F, *n* = 5 rats, 15 cells). Scale bar: 100 pA/100 ms. ***C***, ***D***, Representative NMDAR-mediated eEPSCs (top) in NAc core MSNs and mean input-output relationship (bottom) of NMDAR-mediated eEPSCs in response to incremental electrical stimulations showing the similar strength of NMDAR-mediated synaptic responses between saline and methamphetamine groups in males (***C***, Bonferroni test, *p* > 0.05: Meth-M, *n* = 8 rats, 17 cells; male saline, *n* = 5 rats, 19 cells) and females (***D***, Bonferroni test, *p* > 0.05; Meth-F, *n* = 7 rats, 10 cells; Sal-F, *n* = 5 rats, 15 cells). Scale bar: 200 pA/100 ms. ***E***, Representative traces (top) and time course (bottom left) of NMDAR-eEPSCs at −40 mV before and during application of TK30 (30 μm), a GluN3 NMDAR antagonist. Quantification (right) of average NMDAR-eEPSC amplitudes before and after TK30 treatment showing no effect of TK30 on NMDAR-eEPSCs and no difference between methamphetamine and saline rats (Sal, *n* = 3 rats, *n* = 6 cells; Meth, *n* = 3 rats, *n* = 5 cells; two-tailed unpaired *t* test, *p* = 0.7042). Scale bar: 50 pA/100 ms. ***F***, Representative traces (top) and time course (left) of NMDAR-eEPSCs at +40 mV before and during application of Ifenprodil (5 μm), a GluN2B NMDAR antagonist. Ifenprodil reduced NMDAR-mediated eEPSCs to a similar relative degree in MSNs from saline and methamphetamine rats. Summary graph (right) showing similar ifenprodil sensitivity between saline and methamphetamine rats (Sal, *n* = 6 rats, 14 cells; Meth, *n* = 6 rats, 8 cells; two-tailed unpaired *t* test, *p* = 0.0903). Scale bar: 100 pA/100 ms. Data are presented as mean ± SEM.

At least one week after cue-induced seeking tests, a subset of male and female rats was used for whole-cell patch-clamp recordings. This subset did not differ in methamphetamine infusions from the total group of rats studied (see Results). We waited at least a week before recording to eliminate any possible small effect of novelty or stress related to the seeking test, with the majority of rats recorded between one and five weeks after the test. The exact interval between the seeking test and slice preparation is unlikely to influence experimental outcomes for several reasons. First, we have not observed effects of a seeking test on AMPAR levels or transmission in the NAc (Conrad et al., 2008; Loweth et al., 2014). Second, we observe the same CP-AMPAR plasticity after methamphetamine incubation regardless of whether rats receive a seeking test. For example, we observed elevated CP-AMPAR levels after methamphetamine incubation (WD7 and WD40–50) in a prior study where no seeking tests were administered to rats destined for electrophysiology (Scheyer et al., 2016), just as the present study revealed CP-AMPAR plasticity in rats that received seeking tests (see Results). Finally, in the methamphetamine rats recorded for this study, we did not observe a significant correlation between the RI and the time elapsed since the seeking test (*r* = 0.257, *p* = 0.1). Each electrophysiological endpoint was evaluated in a minimum of eight cells from five rats, except for confirmatory experiments that used five to six cells from three rats (Fig. 4*E*,*F*). Student’s *t* tests (independent unless otherwise indicated) were used for comparing two groups (Figs. 2*B*, 4*E*,*F*). One-way and two-way ANOVAs were used for comparing multiple groups followed by Tukey’s or Bonferroni *post hoc* multiple comparisons. See Results and legends to Figures 2–4 for details of statistical analysis. Clampfit (v11, Molecular Devices) and GraphPad Prism 9.2 were used for figures and statistics for electrophysiology data.

#### Biochemistry

**Figure 5.**
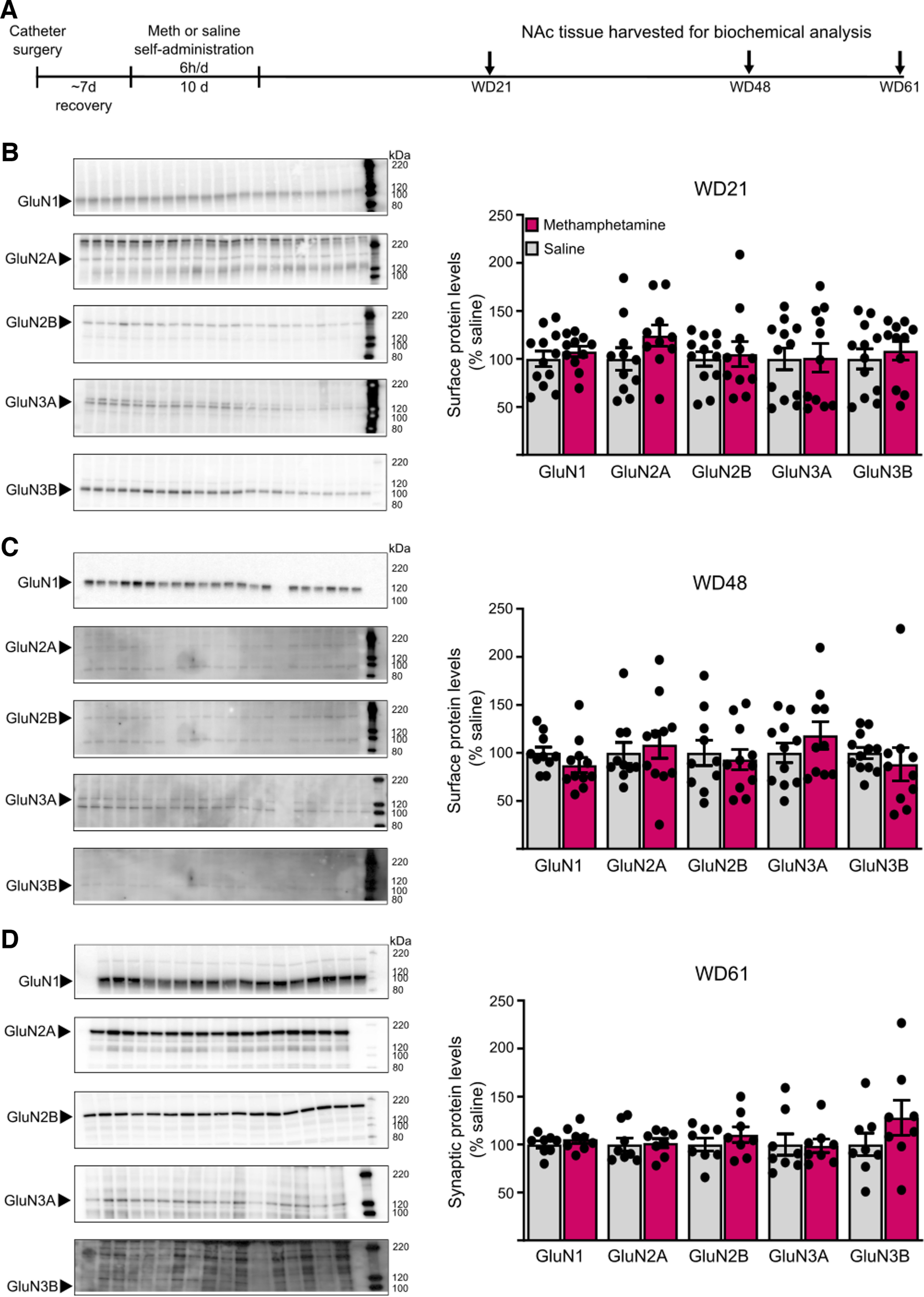
NMDAR subunit protein levels in cell surface and postsynaptic density (PSD) fractions are unchanged after withdrawal from long-access methamphetamine self-administration. ***A***, Male rats received jugular catheters, and underwent methamphetamine or saline self-administration for 10 d. NAc tissue was dissected for analysis of cell surface proteins (***B***, ***C***) or PSD proteins (***D***). ***B***, ***C***, NAc core tissue from methamphetamine and saline rats was biotinylated to label cell surface proteins and immunoblotted as described in Materials and Methods (WD21: saline, *n* = 12; methamphetamine, *n* = 11; WD48: saline, *n* = 11; methamphetamine, *n* = 10). Left, Representative immunoblots of biotinylated tissue at each withdrawal time point for each subunit. Right, Summary data of surface expression of each subunit. No significant change in surface expression of any subunit was detected in methamphetamine rats compared with saline rats on either WD21 (***B***) or WD48 (***C***; *t* tests, *p* > 0.05). There were also no significant changes in total NMDAR subunit levels in homogenates (data summarized in Results). ***D***, Triton-insoluble PSD fractions were prepared from NAc tissue on WD61 as described in Materials and Methods (saline, *n* = 8; methamphetamine, *n* = 8). Left, Representative immunoblots for each subunit. Right, Summary data of PSD levels of each subunit. No significant difference was found for any subunit between methamphetamine and saline rats (*t* tests, *p* > 0.05). For representative immunoblots in ***B–D***, arrows on the left indicate the band analyzed (GluN1 ∼120 kDa, GluN2A ∼180 kDa, GluN2B ∼200 kDa, GluN3A ∼125 kDa, GluN3B ∼100 kDa) and numbers on the right indicate molecular weights (kDa) on the protein ladder. Summary data are presented as percent of average levels in saline animals (mean ± SEM), with dots indicating individual data points. The number of lanes presented on the blots may differ from the number of data points included in the analysis because imperfections in a particular lane prevented analysis. GluN3B bands appear faint in the images but could be selected and analyzed using our gel documentation software. A Shapiro–Wilk test was used to assess normality of data. Parametric data were analyzed using a Student’s *t* test (WD21 surface GluN1, GluN2A, GluN2B, GluN3A; WD48 surface GluN2B, GluN3A; WD61 PSD GluN1, GluN2B, GluN3B, *p* > 0.05), and nonparametric data were analyzed using a Mann–Whitney *U* test (WD21 surface GluN3B; WD48 surface GluN1, GluN2A, GluN3B; WD61 PSD GluN2A, GluN3A, *p* > 0.05).

Biochemical studies were performed before the other studies presented here. Using tissue from male rats, we measured total, cell surface, or synaptic NMDAR subunit protein levels. The smallest group size was eight rats. Student’s independent *t* tests were used when data passed normality tests; in cases of non-normality, Mann–Whitney *U* tests were used to compare groups. See Results and legend to Figure 5 for details of statistical analysis.

## Results

### Self-administration training

Male and female rats self-administered saline or methamphetamine under long-access conditions (Fig. 1*A–F*). Infusions during training (Fig. 1*B*,*C*) were analyzed using a generalized linear mixed model (under a γ distribution) with sex and drug group as between-subject factors and training day (session) as the within-subject factor. There were significant main effects of drug group (*F*_(1,1488)_ = 494.65, *p* < 0.001), session (*F*_(1,1488)_ = 3.048, *p* = 0.001), and sex (*F*_(1,1488)_ = 7.178, *p* = 0.007) and two significant interactions: session × drug group (*F*_(9,1488)_ = 24.736, *p* < 0.001) and drug group × sex (*F*_(9,1488)_ = 9.623, *p* = 0.002). Other interactions were nonsignificant (session × sex, session × drug group × sex; *p* > 0.05). *Post hoc* analyses were performed with sequential Bonferroni adjustment unless otherwise stated. Methamphetamine rats took significantly more infusions than saline rats on days 3–10 (groups compared on each day, all *p* < 0.05; Fig. 1*B*) and increased their drug intake from day 1 to day 10 (*t*_(1488)_ = 3.074, *p* < 0.001), whereas saline rats decreased their intake (*t*_(1488)_ = 4.434, *p* < 0.001; Fig. 1*B*). Males had higher methamphetamine intake on days 6–7 and 9–10 than females (sexes compared on each day, all *p* < 0.05; day 8, *p* = 0.051; Fig. 1*C*). Furthermore, males had higher average daily intake than females (unpaired *t* test, *t*_(1488)_ = 6.221, *p* < 0.001; Fig. 1*E*).

Nose-pokes during self-administration training were analyzed using a generalized linear mixed model (under a γ distribution) with training day (session) and hole (active and inactive) as the within-subject factors, and sex and drug group as between-subject factors. There were significant main effects of drug group (*F*_(1,2917)_ = 121.367, *p* < 0.001), hole (*F*_(1,2917)_ = 241.92, *p* < 0.001), and session (*F*_(9,2917)_ = 4.719, *p* < 0.001), as well as a significant interaction of session × drug group × hole (*F*_(9,2917)_ = 2.222, *p* = 0.018) and drug group × hole (*F*_(9,2917)_ = 58.051, *p* < 0.001). Methamphetamine rats performed more nose-pokes in the active hole throughout training (*p* > 0.05 for active/inactive comparison on each day). Saline rats also discriminated between active/inactive holes (*p* > 0.05 on each day), as we sometimes observe (Werner et al., 2015), although they performed fewer active hole nose-pokes than methamphetamine rats on days 3–10 (*p* < 0.05 on each day; Fig. 1*D*). In contrast to infusion data, no sex differences were detected in active or inactive hole nose-pokes during training [no main effect of sex, sex × session interaction, or sex × hole interaction; *p* > 0.05] or average daily active hole nose-pokes (Mann–Whitney *U* = 2028, *p* = 0.24; Fig. 1*F*). In summary, methamphetamine rats of both sexes learned to discriminate between active and inactive holes and increased their intake throughout training, although males took more methamphetamine.

### Time course of incubation of methamphetamine craving

Using the same methamphetamine regimen in male rats, we previously demonstrated significantly higher levels of cue-induced seeking by WD7, compared with WD1, that are maintained through WD45 (Scheyer et al., 2016; Murray et al., 2019). To extend these findings to females and longer withdrawal times, each rat received a single cue-induced seeking test on either WD1, WD7, WD30, or WD100 (Fig. 1*A*). More rats were tested on WD1. These rats were all indistinguishable from each other (same strain, same vendor, same methamphetamine regimen, same operant chambers, same lab personnel) and their drug intake during self-administration was indistinguishable from other rats in this study, so all were included. During seeking tests, responding in the previously active hole delivered the light cue but no drug infusion; this was our operational measure of cue-induced drug seeking. Nose-pokes in the previously active hole were analyzed using a Generalized Linear Model (under a γ distribution) with WD and sex as between-subject factors, revealing a significant main effect of WD (Wald χ^2^_(3)_ = 34.473, *p* < 0.001) but not sex (Wald χ^2^_(1)_ = 0.295, *p* = 0.587) and no significant WD × sex interaction (Wald χ^2^_(3)_ = 0.354, *p* = 0.949). Sequential Bonferroni *post hoc* analyses indicated a significant increase in cue-induced seeking from WD1 to WD7 (*t*_(1)_ = −3.574, *p* = 0.025), from WD1 to WD30 (*t*_(1)_ = −3.180, *p* = 0.029), and from WD1 to WD100 (*t*_(1)_ = −7.978, *p* < 0.001). WD100 seeking was significantly higher than both WD7 (*t*_(1)_ = −3.741, *p* = 0.029) and WD30 (*t*_(1)_ = −3.288, *p* = 0.018; Fig. 1*G*). Male and female rats did not differ in the number of active nose-pokes on any WD (WD100, *t*_(1)_ = −0.784; WD30, *t*_(1)_ = −0.128; WD7, *t*_(1)_ = 0.165; WD1, *t*_(1)_ = −0.291; all *p* = 1.000; Fig. 1*H*). Inactive hole nose-pokes, analyzed as described for active hole nose-pokes, did not change significantly as a function of WD and did not show sex differences (no significant main effect of WD, sex, or WD × sex interaction, *p* > 0.05; Fig. 1*G*). Finally, our results, although limited, do not suggest a difference between estrous and nonestrous female rats in active hole nose-pokes (Fig. 1*H*).

In summary, male and female rats did not differ in the timing of incubation of methamphetamine craving. While craving plateaued between WD7 and WD30, matching previous reports (Scheyer et al., 2016; Adhikary et al., 2017), it appears to further intensify between WD30 and WD100.

### Rats for electrophysiological studies

A subset of rats from behavioral studies, selected randomly based on their availability on recording days, was used for electrophysiological studies of NAc core MSNs of male and female rats. As shown for all rats (Fig. 1), this subset exhibited an increase in average daily infusions over training (*t*_(520)_ = 9.619, *p* < 0.001) and greater overall intake in males than females (*t*_(540)_ = 3.549, *p* = 0.001).

### Membrane properties of NAc core MSNs

To assess intrinsic neuronal properties, we measured resting membrane potential (RMP), rheobase (a measure of the threshold current required to initiate an action potential) in response to depolarizing ramp currents (1 s, 0–500 pA, ramp), excitability (the relationship between injected current strength and the subsequent quantity of spikes; 0–600 pA, Δ50 pA, 500-ms duration steps), and input resistance (R_input_; −150–25 pA, Δ25 pA subthreshold steps). Focusing first on saline controls (WD30–48), depolarizing step currents induced more action potentials in MSNs from female rats than males at injected currents ranging from 300 pA to 550 pA (two-way RM ANOVA, sex × injected current interaction, *F*_(12,660)_ = 7.711, *p* < 0.0001; sex, *F*_(1,55)_ = 10.07, *p* = 0.0025; injected current, *F*_(12,660)_ = 319.7, *p* < 0.0001; Fig. 2*A*). This higher excitability was associated with lower spike thresholds (*t*_(54)_ = 2.906, *p* = 0.0053; Fig. 2*B*) and smaller rheobase currents (two-way ANOVA, sex × group interaction, *F*_(2,130)_ = 6.712, *p* = 0.0017; sex, *F*_(1,130)_ = 5.175, *p* = 0.0245; group, *F*_(2,130)_ = 3.236, *p* = 0.0425; male saline vs female saline, q_(1,43)_ = 5.818, *p* = 0.0010; Fig. 2*C*). Males and females were similar with regard to passive properties such as RMP and R_input_ (all *p* > 0.05; Fig. 2*D–F*).

To determine whether methamphetamine withdrawal alters these properties, we compared saline rats (WD30–48), methamphetamine rats on WD30–75, and methamphetamine rats on WD100–135. While the range of withdrawal days examined for saline rats was limited compared with methamphetamine rats, we have never observed time-dependent changes in MSN properties in saline rats recorded between WD1 and ∼WD70 over >10 years of recordings. In male rats, MSNs from both methamphetamine groups showed a leftward shift in the current-spike relationship compared with saline controls indicating increased excitability (two-way RM ANOVA: injected current × drug/withdrawal history interaction, *F*_(24,792)_ = 6.031, *p* < 0.0001; main effect of drug/withdrawal history, *F*_(2,66)_ = 7.104, *p* = 0.0016; main effect of injected current, *F*_(12,792)_ = 429.6, *p* < 0.0001; Fig. 2*G*), although the shift was significant across more stimulus intensities for the methamphetamine WD30–75 group than the methamphetamine WD100–135 group (Fig. 2*G*). The methamphetamine WD30–75 group showed decreased rheobase currents [Tukey’s test after two-way ANOVA (see above), saline vs methamphetamine WD30–75, q_(1,53)_ = 4.423, *p* = 0.009; Fig. 2*C*], but this was not observed for the methamphetamine WD100–135 group (saline vs methamphetamine WD100–135, q_(1,46)_ = 1.153, *p* > 0.5; Fig. 2*C*). Methamphetamine withdrawal did not alter passive properties of MSNs in males (all *p* > 0.05; Fig. 2*D–F*).

In female rats, however, methamphetamine and saline MSNs exhibited similar excitability (two-way RM ANOVA, no main effect of drug/withdrawal history, *F*_(2,74)_ = 3.095, *p* > 0.05; Fig. 2*H*) and rheobase currents (Tukey’s test, saline vs methamphetamine WD30–75, q_(1,47)_ = 2.386, *p* = 0.5429; saline vs methamphetamine WD100–135, q_(1,33)_ = 3.563, *p* = 0.1260; Fig. 2*C*). Passive membrane properties were also not altered (all *p* < 0.05; Fig. 2*D–F*). Notably, two-way ANOVA (4 groups: male and female methamphetamine WD30–75 and WD100–130 rats in Fig. 2*G*,*H*) revealed a significant effect of injected currents (*F*_(12,1032)_ = 610.4, *p* < 0.0001) and a significant interaction between sex/withdrawal history and injected currents (*F*_(36,1032)_ = 2.687, *p* < 0.001) but no significant main effect of sex/withdrawal history (*F*_(3.86)_ = 1.156, *p* = 0.331). Tukey’s *post hoc* tests also showed no differences in excitability between male and female rats across methamphetamine groups (*p* > 0.05).

Overall, these data indicate some differences in membrane properties and neuronal excitability between male and female rats in the saline group, as found by others (see Discussion), but our methamphetamine regimen affects MSNs from male rats such that male and female rats exhibit similar membrane properties and excitability after incubation of methamphetamine craving.

### Contribution of CP-AMPARs to synaptic transmission

We previously compared NAc core MSNs from male rats on WD1, WD2–4, WD7–8, and WD40–50 from methamphetamine self-administration and found significant CP-AMPAR elevation, relative to WD1, on WD7–8 and WD40–50 (Scheyer et al., 2016). To examine longer withdrawal times in males and determine whether similar plasticity occurs in females, we generated I–V curves for the AMPAR-mediated evoked EPSC (eEPSC) in male and female methamphetamine rats to assess inward rectification, a hallmark of CP-AMPARs. We compared WD15–35, WD40–75, and WD100–135 methamphetamine groups and a WD30–61 saline group.

In males, saline MSNs exhibited a linear I–V relationship, whereas all methamphetamine groups showed an elevated rectification index or RI [eEPSC_−70mV_/(−70-E_rev_)]/[eEPSC_+40mV_/(+40-E_rev_)] (two-way RM ANOVA, Vh × drug/withdrawal history interaction, *F*_(21,721)_ = 18.30, *p* < 0.0001; main effect of drug/withdrawal history, *F*_(3,103)_ = 10.58, *p* < 0.0001; main effect of Vh, *F*_(3,285)_ = 27 264, *p* < 0.0001; Fig. 3*A*). *Post hoc* analysis (Tukey) revealed a higher RI in methamphetamine WD15–35 rats (q_(1,55)_ = 8.941, *p* < 0.0001) and methamphetamine WD40–75 rats (q_(1,52)_ = 9.374, *p* < 0.0001) relative to saline controls (Fig. 3*B*). Male WD100–135 methamphetamine rats still showed an increased RI compared with saline rats (q_(1,58)_ = 3.808, *p* < 0.05) but their RI was lower than that of methamphetamine WD15–35 rats (q_(1,57)_ = 5.319, *p* < 0.01) or WD40–75 rats (q_(1,54)_ = 5.867, *p* < 0.001; Fig. 3*B*).

To confirm these results, eEPSCs were measured before and after bath application of the selective CP-AMPAR antagonist 1-naphthylacetyl spermine trihydrochloride (NASPM; 100 μm; Fig. 3*C*). Male methamphetamine rats displayed a significant NASPM-induced reduction in eEPSC amplitude, whereas saline rats did not (two-way RM ANOVA, time × drug/withdrawal history interaction, *F*_(225,2700)_ = 2.624, *p* < 0.0001; main effect of drug/withdrawal history, *F*_(3,36)_ = 11.84, *p* < 0.001; main effect of time, *F*_(75,2700)_ = 13.20, *p* < 0.0001; Fig. 3*C*). Mean eEPSC amplitudes, measured 15–20 min after NASPM application and expressed as percent of baseline, were significantly reduced in all male methamphetamine groups compared with saline controls (one-way ANOVA, *F*_(3,35)_ = 17.43, *p* < 0.0001; Tukey’s multiple comparisons vs saline: methamphetamine WD15–35: q_(1,20)_ = 7.287, *p* < 0.0001; methamphetamine WD40–75: q_(1,17)_ = 9.968, *p* < 0.0001; methamphetamine WD100–135: q_(1,18)_ = 5.243, *p* < 0.01; Fig. 3*D*). The male methamphetamine WD15–35 and WD40–75 groups did not differ (q_(1,21)_ = 3.441, *p* > 0.05) but NASPM sensitivity was lower in WD100–135 rats than WD40–75 rats (q_(1,19)_ = 5.129, *p* < 0.01; Fig. 3*D*), consistent with a tendency toward lower RI values in the WD100–135 group (Fig. 3*B*).

Similar to males, female saline rats exhibited a linear I–V curve, whereas female methamphetamine rats displayed significant inward rectification at the three withdrawal times studied (two-way RM ANOVA, Vh × drug/withdrawal history interaction, *F*_(21,847)_ = 18.09, *p* < 0.0001; main effect of drug/withdrawal history, *F*_(3,121)_ = 8.062, *p* < 0.0001; main effect of Vh, *F*_(7,847)_ = 23 544, *p* < 0.0001; Fig. 3*E*). Quantification of the RI indicated a marked increase in female methamphetamine rats (one-way ANOVA, *F*_(3,119)_ = 21.82, *p* < 0.0001; Tukey’s multiple comparisons vs saline: methamphetamine WD15–35: q_(1,63)_ = 10.59, *p* < 0.0001; methamphetamine WD40–75, q_(1,55)_ = 9.329, *p* < 0.0001; methamphetamine WD100–135, q_(1,59)_ = 7.516, *p* < 0.0001; Fig. 3*F*). Furthermore, MSNs from female methamphetamine rats displayed reduced eEPSC amplitude after bath application of NASPM, whereas female saline rats showed no reduction (two-way RM ANOVA, time × drug/withdrawal history interaction, *F*_(225,3450)_ = 2.922, *p* < 0.0001; main effect of drug/withdrawal history, *F*_(3,46)_ = 12.12, *p* < 0.0001; main effect of time, *F*_(75,3450)_ = 11.92, *p* < 0.0001; Fig. 3*G*). Analysis of mean eEPSC amplitude after NASPM, expressed as percent of baseline, indicated a significant effect of NASPM in MSNs from female methamphetamine rats across withdrawal compared with saline rats (one-way ANOVA, *F*_(3,46)_ = 16.46, *p* < 0.0001; Tukey’s multiple comparisons vs saline: methamphetamine WD15–35, q_(1,27)_ = 7.941, *p* < 0.0001; methamphetamine WD40–75, q_(1,20)_ = 8.660, *p* < 0.0001; methamphetamine WD100–135, q_(1,21)_ = 8.666, *p* < 0.0001; Fig. 3*H*).

Direct comparison of male and female rats indicated no significant effect of sex on CP-AMPAR-mediated synaptic plasticity in NAc core, measured as either RI or NASPM sensitivity, after withdrawal from methamphetamine (Fig. 3*I*,*J*; replotted for ease of comparison from Fig. 3*B*,*D*,*F*,*H*). Briefly, Tukey’s tests after two-way ANOVOA revealed no differences in RI values between males and females in saline (q_(1,55)_ = 2.627, *p* = 0.5813), methamphetamine WD15–35 (q_(1,63)_ = 1.689, *p* = 0.9329), methamphetamine WD40–75 (q_(1,52)_ = 2.893, *p* = 0.4537) or methamphetamine WD100–135 (q_(1,62)_ = 0.9917, *p* = 0.9969) groups. Likewise, there were no differences in NASPM sensitivity between males and females (same group comparisons: q_(1,17)_ = 1.371, *p* = 0.9775; q_(1,30)_ = 1.549, *p* = 0.9560; q_(1,20)_ = 3.079, *p* = 0.3763; q_(1,22)_ = 1.936, *p* = 0.8686). In summary, similar CP-AMPAR plasticity occurs in both sexes and persists through WD100–135.

### NMDAR synaptic transmission in NAc core MSNs after methamphetamine withdrawal

NMDARs are tetramers that include two copies of the obligatory GluN1 subunit along with GluN2 (GluN2A-D) and/or GluN3 (GluN3A or GluN3B) subunits (Hansen et al., 2018; Stroebel et al., 2018). After incubation of cocaine craving, we detected enhanced NMDAR-mediated EPSCs in NAc core MSNs of male rats at positive and negative holding potentials attributable to GluN2B-containing NMDARs and GluN3-containing NMDARs, respectively (Christian et al., 2021). Shifts in inclusion of GluN2B and GluN3 can significantly alter synaptic transmission and plasticity (Pachernegg et al., 2012; Perez-Otano et al., 2016; Turner et al., 2018).

To determine whether similar plasticity occurs in NAc core MSNs after methamphetamine incubation, we recorded electrically-evoked NMDAR-mediated EPSCs in MSNs from male and female rats (WD19–53) at different holding potentials to generate I–V curves. No differences were found between: male saline and methamphetamine groups (two-way ANOVA, Vh × drug history interaction, *F*_(7,259)_ = 0.8452, *p* = 0.5508; drug history, *F*_(1,37)_ = 1.725, *p* = 0.1972; Fig. 4*A*), female saline and methamphetamine groups (two-way ANOVA, Vh × drug history interaction, *F*_(7,168)_ = 0.6618, *p* = 0.7041; drug history, *F*_(1,24)_ = 0.1380, *p* = 0.7135; Fig. 4*B*), or male and female saline rats (compared between Fig. 4*A* and *B*). Furthermore, NMDA input-output relationships indicate no difference in the strength of NMDAR-mediated synaptic transmission between saline and methamphetamine groups in either male rats (two-way ANOVA, drug history, *F*_(1,34)_ = 0.1283, *p* = 0.7224; intensity, *F*_(1,38)_ = 116.4, *p* < 0.0001; drug history × intensity interaction, *F*_(9,306)_ = 2.008, *p* = 0.0381, Bonferroni’s test, *p* > 0.5; Fig. 4*C*) or female rats (two-way ANOVA, drug history, *F*_(1,23)_ = 0.6611, *p* = 0.4245; intensity, *F*_(1,29)_ = 140.4, *p* < 0.0001, drug history × intensity interaction, *F*_(9,207)_ = 0.2246, *p* = 0.9907, Bonferroni’s test, *p* > 0.5; Fig. 4*D*). Finally, we applied TK30 (Kvist et al., 2013) and ifenprodil to selectively block GluN3-containing and GluN2B-containing NMDARs, respectively. TK30 did not affect NMDAR-mediated eEPSC amplitude (−40-mV holding potential) in either saline or methamphetamine rats (males and females combined; Fig. 4*E*), consistent with negative results in male saline rats in our previous study (Christian et al., 2021). Ifenprodil decreased the NMDAR-mediated EPSC (40-mV holding potential) to the same relative degree in MSNs from saline and methamphetamine groups (males and females combined; Fig. 4*F*). Ifenprodil has previously been shown to decrease the NMDAR EPSC in MSNs from male drug-naive animals (Chapman et al., 2003; Kasanetz and Manzoni, 2009; Christian et al., 2021).

Complementing electrophysiological work, we used NAc tissue from previously generated cohorts of male saline and methamphetamine rats (same self-administration regimen; see Materials and Methods for more details) to measure NMDAR subunit levels. These rats were killed over a range of withdrawal times (WD21–61 depending on the experiment; Fig. 5*A*) that overlaps with the range used in electrophysiological studies of NMDAR transmission described above (WD19–53). Comparing saline and methamphetamine groups killed on WD21 or WD48 for surface biotinylation studies, we found no group differences in cell surface NMDAR subunit levels in cell surface fractions at either time point (Fig. 5*B*,*C*). We also found no group differences in homogenates that were the starting material for isolating the biotinylated fraction (data from methamphetamine groups are presented as mean percent of corresponding saline groups ± SEM; WD21: GluN1 110.0 ± 4.0, GluN2A 109.6 ± 11.0, GluN2B 94.3 ± 7.7, GluN3A 106.3 ± 5.2, GluN3B 105.3 ± 6.0; WD48: GluN1 94.5 ± 7.6, GluN2A 84.5 ± 10.3, GluN2B 99.0 ± 13.4, GluN3A 141.0 ± 38.7, GluN3B 90.9 ± 7.6; *p* > 0.05 vs respective saline group in all cases). In an additional cohort of saline and methamphetamine rats killed on WD61, we found no group differences in NMDAR subunit levels in postsynaptic density fractions (Fig. 5*D*). Because electrophysiological results described above did not reveal sex differences or effects of methamphetamine on NMDAR transmission and no effects of methamphetamine were found in these biochemical studies of NMDAR subunits in male rats, we did not conduct additional biochemical studies in female rats.

## Discussion

We compared cue-induced craving and electrophysiological properties of NAc core MSNs in male and female rats across 100+ days of forced abstinence.

### Methamphetamine self-administration and incubation of craving

We found that males self-administered more methamphetamine than females during the latter half of self-administration training. Greater intake in males was also found in studies that used a similar dose (0.08 or 0.1 mg/kg/infusion) and initiated training under long-access conditions (Ruda-Kucerova et al., 2015; Daiwile et al., 2019, 2021). At lower doses, greater intake by females (0.02 mg/kg; Reichel et al., 2012) or no sex difference (0.05 mg/kg; Pena-Bravo et al., 2019) was reported. In studies that used 0.1 mg/kg but in which social or food self-administration training (Venniro et al., 2017, 2018) or short-access methamphetamine training (Everett et al., 2020) preceded long-access self-administration, no sex differences were observed. Thus, many aspects of experimental design influence methamphetamine intake in male versus female rats. In a human study, there was no sex difference in average dose of methamphetamine used after controlling for age and BMI; before accounting for these factors, males took higher doses (He et al., 2013).

Despite differences in intake, male and female rats exhibited similar levels of cue-induced craving across 100 d of forced abstinence. In agreement with our results, all studies cited above that employed long-access self-administration found similar incubation in males and females (Venniro et al., 2017; Daiwile et al., 2019, 2021; Everett et al., 2020). A study using 90-min sessions found higher drug seeking in females on WD14 but incubation was not demonstrated (Ruda-Kucerova et al., 2015). In studies of cocaine incubation, females exhibit higher seeking but this is driven by females in estrus (Kerstetter et al., 2008; Nicolas et al., 2019; Corbett et al., 2021). In our study, the number of females in estrus during seeking tests was too low for reliable conclusions, although no estrus/nonestrus difference was obvious. In other studies, methamphetamine-primed reinstatement (Reichel et al., 2012; Cox et al., 2013) and cue-induced methamphetamine seeking (Ruda-Kucerova et al., 2015) did not depend on estrous cycle stage.

In both males and females, the rising phase of incubation corresponded to previous reports: significant incubation by WD7 that plateaus through WD30 (Scheyer et al., 2016; Adhikary et al., 2017). Surprisingly, seeking was further increased by WD100. Before the present work, the latest withdrawal time examined was WD51 (males only; Shepard et al., 2004). Future studies should determine when incubation wanes, although for cocaine incubation this depends on the paradigm (Lu et al., 2004; Madangopal et al., 2019).

How well do these findings map onto the clinical literature? While studies of craving and relapse during abstinence in methamphetamine users are sparse, men and women generally exhibit more similarities than differences (see Introduction). The only study to specifically examine incubation of methamphetamine craving in humans was conducted in male inpatients; while baseline craving fell during abstinence, cue-induced craving increased over the first three months of abstinence and then decreased at six-month and one-year timepoints (G. Wang et al., 2013). It is important from the standpoint of translational relevance that incubation of methamphetamine craving can be demonstrated in both species, but comparing specifics of its timing between species is problematic because of the many differences in nature/duration of methamphetamine exposure and in life history before and after methamphetamine exposure in rodent incubation studies versus this human study. For example, the rats returned to the home cage while the humans were in a clinical setting. Furthermore, rats and humans may differ in the capacity for brain and behavioral recovery.

### Electrophysiological properties of NAc core MSNs

Prior work has demonstrated some sex differences in NAc core MSNs. Focusing on work in adults, females show greater spine density and more large spines (Forlano and Woolley, 2010; Wissman et al., 2011, 2012) and higher mEPSC frequency with no change in paired-pulse ratio (Wissman et al., 2011). Furthermore, intrinsic electrophysiological and excitatory synaptic properties change across the estrous cycle and are regulated by sex hormones (Proano et al., 2018, 2020; Alonso-Caraballo and Ferrario, 2019; Krentzel et al., 2019; Proano and Meitzen, 2020). Similar findings are reported in NAc core of prepubertal rats (Cao et al., 2016) but not mice (Cao et al., 2018; for review, see Krentzel and Meitzen, 2018).

Consistent with sex differences in MSN properties, among our saline controls, MSNs from females exhibited greater excitability associated with lower spike thresholds and smaller rheobase currents, although males and females were similar for passive membrane properties. Interestingly, the sex difference in excitability was eliminated after methamphetamine withdrawal because of increased excitability in MSNs from males. As both sexes exhibited incubation, this may suggest that the sex-dependent excitability changes are not related to incubation (but see next paragraph for speculation regarding a possible contribution in males). A limitation is that we did not make excitability measures on WD1, so we cannot say whether they track with incubation although they are useful in further characterizing the state of MSNs during the withdrawal period when CP-AMPAR elevation and incubation are manifest. A more general limitation is that recordings in saline rats were conducted over a shorter range of withdrawal times (WD30–61) than methamphetamine rats (WD15–135), so we cannot rule out effects of aging on our measures. Arguing against this, we have never observed differences in properties of saline rats across WD1 through ∼WD70 in 10+ years of prior studies, and time-dependent effects of methamphetamine exposure sometimes differed in males and females suggesting these effects are not simply because of aging (although it is possible aging has different cellular consequences in males and females).

Consistent with our prior work in saline rats (Conrad et al., 2008; Scheyer et al., 2016; Kawa et al., 2022), male and female saline rats exhibited linear current–voltage relationships and no significant sensitivity to NASPM, indicating low CP-AMPAR levels. In contrast, we observed inward rectification and NASPM sensitivity, indicative of CP-AMPAR transmission, in male and female methamphetamine rats on WD15–35, WD40–75, and WD100–135. In males, we previously examined earlier withdrawal times after the same methamphetamine regimen; compared with saline controls, CP-AMPAR levels were unchanged on WD1, trending upward on WD2–4, and significantly elevated on WD7–8, paralleling incubation of craving (Scheyer et al., 2016). We also showed that activation of CP-AMPARs is necessary for expression of incubated craving on WD45 (Scheyer et al., 2016). Our working model is that strengthening of NAc synapses via insertion of high-conductance CP-AMPARs enables MSNs to respond more robustly to glutamate release in response to cue presentation, resulting in stronger cue-induced methamphetamine seeking. We hypothesize that CP-AMPAR upregulation continues to be required for expression of methamphetamine incubation through WD100 but this remains to be tested. It is interesting in this regard that incubation remained robust on WD100, whereas CP-AMPAR levels in males (but not females) were modestly reduced in the WD100–135 methamphetamine group compared with methamphetamine rats studied on WD15–35 or WD40–75. It should be emphasized, however, that the CP-AMPAR levels in WD100–135 male rats remained significantly elevated compared with saline controls and were not significantly different from females assessed on WD100–135. We hypothesize that the CP-AMPAR upregulation that exists on WD100–135 is sufficient to support incubation in males although it is also possible that additional adaptations in the NAc (e.g., the persistent enhancement of excitability in males, measured with current-spike relationships) or other brain regions combine with this CP-AMPAR elevation to support incubation. These possibilities can be tested in future studies. Additional challenges are to extend our findings in a cell-type and pathway-specific manner and understand how AMPAR (Scheyer et al., 2016) and dopamine (Rossi et al., 2020) transmission in NAc core interact to mediate expression of incubation.

Our results also enable comparisons between cocaine and methamphetamine incubation. While they have in common a necessary role for CP-AMPAR upregulation in NAc core (Conrad et al., 2008; Scheyer et al., 2016) and both sexes show CP-AMPAR upregulation after cocaine (Kawa et al., 2022) and methamphetamine (present results), cocaine incubation is associated with altered NMDAR transmission in the NAc core (Christian et al., 2021) while methamphetamine incubation is not. Cocaine and methamphetamine incubation also differ with regard to group I metabotropic glutamate receptor involvement (Murray et al., 2019, 2021).

A prior study compared excitatory synaptic transmission in NAc core MSNs (males) 8 d after long-access methamphetamine self-administration (seven 1-h daily sessions followed by 14 daily 6-h sessions; yoked saline controls). Field recordings revealed an increase in release probability and the input-output function in methamphetamine rats, while whole-cell recordings found an increase in spontaneous EPSC frequency, but no change in spontaneous EPSC amplitude or the AMPA/NMDA ratio; incubation of craving was not tested (Mishra et al., 2017). Interestingly, whole-cell recordings in prelimbic prefrontal cortex of male and female rats found sex differences in spontaneous excitatory activity in drug-naive rats and in NMDAR transmission after 8–14 d of withdrawal from long-access methamphetamine self-administration (Mishra et al., 2017; Pena-Bravo et al., 2019). How these adaptations combine with those observed in the present study to influence reward-related circuitry remains to be tested.

In conclusion, it is striking that a relatively short period of methamphetamine exposure (10 d) resulted in incubated craving and strengthening of excitatory synaptic transmission in the NAc core (via CP-AMPAR upregulation) that persisted for 100+ days in both sexes.

## Synthesis

Reviewing Editor: Tod E Kippin, University of California Berkeley Decisions are customarily a result of the Reviewing Editor and the peer reviewers coming together and discussing their recommendations until a consensus is reached. When revisions are invited, a fact-based synthesis statement explaining their decision and outlining what is needed to prepare a revision will be listed below. The following reviewer(s) agreed to reveal their identity: Karen Szumlinski. Note: If this manuscript was transferred from JNeurosci and a decision was made to accept the manuscript without peer review, a brief statement to this effect will instead be what is listed below.

I am happy to inform you that I have received two independent reviews both recommending that your manuscript is suitable or very nearly suitable for publication. Reviewer 1 did have some minor issues that will need to be address before finalizing the decision. Please let me know if you require any assistance in dealing with the comments.

Below are the comments of the reviewers:

Reviewer 1:

In this manuscript, with contains reviews transferred from another journal, the authors describe a series of studies aimed at examining the incubation of methamphetamine craving in rats and its correspondence with NAc medium spiny neuron physiology, CP-AMPA responses, and NMDAR subunit expression. The key finding presented is that cue-evoked meth seeking and accompanying changes in NAc MSNs persist 100 days into forced abstinence, extending prior findings by the authors and other laboratories examining this phenomenon for shorter periods of abstinence.

There are some methodological issues with the study, primarily stemming from the senior author’s change of institution which lead to conducting studies at separate universities, using tissue homogenates from prior cohorts of animals, and not all measures being obtained and all time points and in both sexes. While these issues impart some limitations, it is this reviewer’s opinion that there is enough cohesion across the data sets can be interpreted in a meaningful way.

I have the following several items of minor concern that are in need of clarity:

1) cefazolin can alter brain levels of GLT-1 which can therefore affect glutamate homeostasis. Can the authors comment on whether the dosing and/or frequency of this antibiotic might have altered glutamate transmission and therefore the experimental results? Theoretically such changes would have also occurred in saline animals, but it is important to note this possibility.

2) line 134 - please clarify whether animals were single housed for the duration of the experiment from this point on.

3) lines 151-158 - were any timeouts implemented during the seeking tests?

4) lines 171, 309 and 313 - the authors state that at least one week elapsed between the seeking test and obtaining tissue for electrophysiology. What was the range of this duration? This seems critical given the time dependency of the changes being measured.

5) line 247 - one of the “0.5%” mentions could be deleted here

6) line 604 - instead of just referring to the Introduction here, it would be helpful to insert relevant references previously cited.

Reviewer 2:

I have read the transferred article authored by Funke, Hwang et al and found the report to be highly responsive to the prior round of review. I agree with the authors that this report contains a significant amount of new data, to include an examination of a 100-day withdrawal time-point. I particularly appreciated their efforts towards reducing the sex-bias of our current knowledge base regarding the physiological and biochemical bases of incubated drug-craving by conducting a large number of studies using females. I have no further suggestions to improve upon this report and I recommend the paper be accepted in its current form.
